# Triptolide attenuates lipopolysaccharide-induced inflammatory responses in human endothelial cells: involvement of NF-κB pathway

**DOI:** 10.1186/s12906-019-2616-3

**Published:** 2019-08-02

**Authors:** Chundong Song, Youping Wang, Lin Cui, Fengna Yan, Si Shen

**Affiliations:** Central Laboratory and Department of Pediatrics Division of Cardiology First Affiliated Hospital, Henan University of Traditional Chinese Medicine, Zhengzhou, 450000 China

**Keywords:** Triptolide, Nuclear factor-κB, Endothelial cells, Inflammation, Lipopolysaccharide

## Abstract

**Background:**

Endothelial cell inflammation is a central event in the pathogenesis of numerous cardiovascular diseases, including sepsis and atherosclerosis. Triptolide, a principal bioactive ingredient of Traditional Chinese Medicine *Tripterygium wilfordii* Hook.F., displays anti-inflammatory actions in vivo. However, the mechanisms underlying these beneficial effects remain undetermined. The present study investigated the effects and possible mechanisms of triptolide on lipopolysaccharide (LPS)-induced inflammatory responses in human umbilical vein endothelial cells (HUVECs).

**Methods:**

The effects of triptolide on the LPS-induced production and expression of inflammatory molecules, monocyte adhesion and activation of nuclear factor (NF)-κB pathway were examined in cultured HUVECs.

**Results:**

In cultured HUVECs, pre-treatment with triptolide dose-dependently attenuated LPS-induced cytokine and chemokine production, adhesion molecule expression and monocyte adhesion. Mechanistically, triptolide was found to dose-dependently inhibit the LPS-induced increases in the DNA binding activity of NF-κB p65 associated with attenuating IκBα phosphorylation and its degradation. Additionally, the present study revealed that triptolide inhibited LPS-triggered NF-κB transcriptional activation in a dose-dependent manner.

**Conclusions:**

The results of the present study indicated that triptolide suppresses the inflammatory response of endothelial cells possibly via inhibition of NF-κB activation.

**Electronic supplementary material:**

The online version of this article (10.1186/s12906-019-2616-3) contains supplementary material, which is available to authorized users.

## Background

It is well accepted that inflammation is involved in the development of cardiovascular diseases [1, 2]. Inflammatory response is the most common characteristic of numerous cardiovascular diseases, including atherosclerosis and hypertension [1–3]. Endothelial dysfunction is a major contributor to the development of various pathological inflammatory conditions [3, 4]. Under these pathological conditions, pro-inflammatory cytokines and chemokines are produced, and adhesion molecules are expressed in endothelial cells, resulting in leukocyte recruitment [5]. Excessive leukocyte accumulation can accelerate the development of inflammatory damage.

*Tripterygium wilfordii* Hook.F. (TWHF, Leigongteng Family Celastraceae), commonly known as thunder god vine in China, and its preparations are widely used to treat inflammatory and autoimmune diseases, including rheumatoid arthritis, systemic lupus, psoriatic arthritis and nephritis [6–8]. Triptolide, extracted from the roots of the Chinese herb TWHF, is a primary bioactive ingredient of this plant [9, 10], and it possesses a broad-spectrum therapeutic potential due to its immunosuppressive, anti-inflammatory and anti-tumor properties [11, 12]. Although considerable efforts have been made to investigate the effects of triptolide on inflammatory responses in vivo [13, 14], only limited data are available that define the mechanism for triptolide-mediated anti-inflammation.

Nuclear factor (NF)-κB is a pleiotropic transcriptional regulator that has been shown to regulate numerous genes. In addition to cell survival and apoptosis, NF-κB is also involved in the transcriptional regulation of pro-inflammatory cytokines, chemokines, adhesion molecules and numerous other genes associated with inflammation [15, 16]. There is accumulating evidence demonstrating that triptolide inhibited the NF-κB pathway in several cell culture systems [17–19]. However, no data are available that define the effects of triptolide on the NF-κB signaling pathway in the development of inflammation in endothelial cells.

Based on the possibility that certain drugs exert their anti-inflammatory effects by targeting endothelial cells, we hypothesized that triptolide-mediated anti-inflammatory effects are due to its inhibition of endothelial cell-associated inflammatory responses. The present study aimed to determine whether triptolide attenuates endothelial cell-associated inflammatory responses via suppression of the NF-κB signaling pathway in vitro in human umbilical vein endothelial cells (HUVECs).

## Methods

### Chemicals and reagents

Triptolide (molecular weight 360.4, purity ≥98% by HPLC), lipopolysaccharide (LPS), 3-(4,5-dimethylthiazol-2-yl)-2,5-diphenyltetrazolium bromide (MTT), dimethyl sulfoxide (DMSO) and protease inhibitor solution were obtained from Sigma Chemical (St. Louis, MO, USA). RPMI-1640 medium, fetal bovine serum (FBS), penicillin, streptomycin, LipofectAMINE 2000 and carboxyfluorescein diacetate succinimidyl ester were purchased from Invitrogen (Camarillo, CA, USA). The cytokine and chemokine ELISA assay kits were obtained from R&D Systems (Minneapolis, MN, USA). RIPA was supplied by Thermo Scientific (Waltham, MA, USA). A protein assay kit and polyvinylidene difluoride (PVDF) membranes were obtained from Bio-Rad Laboratories (Hercules, CA, USA).

### Media and cell lines

HUVECs were purchased from Lonza (Catalogue No: C2517A, Allendale, NJ, USA) and cultured in EGM-2 Bullet kit medium supplied with growth supplements (Lonza, Allendale, NJ, USA) in a humidified atmosphere at 37 °C with 5% CO_2_. HUVECs were cultured to 80–90% confluence and starved in endothelial basal medium-2 (EBM-2) with no growth supplements for 4 h before the experiment was initiated. Cells used in the experiments were between 3 and 7 passages.

### Triptolide treatment

Triptolide was dissolved in DMSO to a stock concentration of 1 mg/ml. For the experiments, cells were cultured in growth supplement-containing medium. After 24 h of incubation, cells were pre-treated with triptolide at concentrations of 25, 50 or 100 nM in growth supplement-free medium at 37 °C for 1 h, and then incubated with LPS for the indicated times. Cells treated with equal dilutions of DMSO alone served as a control.

### Cell viability assay

Cell viability was evaluated using an MTT colorimetric assay. In brief, HUVECs were cultured in 96-well plates (1 × 10^4^ cells per well) and allowed to adhere overnight. Following treatment with LPS or triptolide at various concentrations for 6, 12 or 24 h, the MTT reagent was added to a final concentration of 0.5 mg/ml and incubated for 4 h. Next, the culture medium with MTT reagent was removed, the water-insoluble formazan crystals were dissolved in DMSO and the absorbance was assayed at 570 nm with a SpectraMax microplate reader (Molecular Devices, Sunnyvale, CA, USA). Cell viability was presented as the change relative to control.

### Cytokine/chemokine assay

HUVECs were cultured in 24-well plates (1 × 10^5^ cells/ml in each well) and treated with LPS, in the presence or absence of triptolide at the indicated concentrations for 6 h. The cell-free supernatant fractions were collected, and the levels of TNF-α, IL-6 and MCP-1 in the culture supernatant were measured by the use of the corresponding ELISA kit, according to the manufacturer’s protocol. The levels of cytokines and chemokines are expressed as pg/ml of culture supernatants.

### Cell-based ELISA assay

The effects of triptolide on the expression of adhesion molecules, including ICAM-1 and VCAM-1, were determined using cell-based ELISA assay as previously described [20]. In brief, HUVECs in 96-well plates were fixed with 4% paraformaldehyde for 5 min and rinsed 3 times with PBS following treatment. The fixed cells were permeabilized with pre-chilled MeOH for 10 min at -4 °C, followed by blocking with PBS containing 1% BSA and 0.2% triton X-100 for 1 h. Subsequently, the cells were incubated with mouse monoclonal antibody against ICAM-1 or mouse monoclonal antibody against VCAM-1 (dilution, 1:100; Santa Cruz Biotechnology, Santa Cruz, CA, USA) at 4 °C for 12 h. Following washing, the cells were incubated with FITC-conjugated anti-mouse secondary antibody (dilution, 1:200; Jackson ImmunoResearch Laboratories, West Grove, PA, USA) for 1 h at room temperature. Incubation with PBS, instead of the primary antibodies, was used as a negative control. The optical density of each well was determined by the use of a SpectraMax microplate reader (Molecular Devices, Sunnyvale, CA, USA) at 485/520 nm. The optical density of the negative control was subtracted as background from that of each well.

### Monocyte adhesion assay

The monocyte adhesion assay was performed as previously described [20]. In brief, HUVECs were cultured in 96-well plates (1 × 10^4^ cells per well) for 12 h and subsequently stimulated with LPS in the presence or absence of triptolide at the indicated concentrations for 6 h. THP-1 cells, a human monocytic cell line (Catalogue No: ATCCTIB-202, ATCC, Manassas, VA, USA), were labeled with 1 μM carboxyfluorescein diacetate succinimidyl ester at 37 °C for 30 min prior to adding the THP-1 to the stimulated HUVECs. The fluorescence-labeled THP-1 cells were rinsed 3–5 times with PBS and resuspended in serum-free RPMI-1640 medium for addition to the stimulated HUVECs. Subsequently, the pre-labeled THP-1 cells (1 × 10^6^ cells per well) were co-cultured with stimulated HUVECs and allowed to attach at 37 °C for 30 min. Next, the cells were rinsed 3–5 times with PBS and incubated with PBS containing 2% Triton X-100 to lyse the THP-1 cells. Cell lysates were analyzed to determine the total fluorescence level by the use of a SpectraMax microplate reader (Molecular Devices, Sunnyvale, CA, USA) at 485/520 nm. In another set of experiments, HUVECs were cultured in 24-well plates (0.5 × 10^5^ cells per well). The fluorescence staining of monocyte adhesion to HUVECs was performed as described earlier. Finally, non-adherent pre-labeled THP-1 cells were removed, and residual cells were rinsed 3 times with PBS and viewed under a fluorescence microscope (DMI 3000B, Leica, Wetzlar, Germany) at 485/535 nm.

### Western blot analysis

HUVECs were lysed in RIPA buffer containing protease inhibitor solution following treatment, and samples were centrifuged at 11,000×g for 20 min to collect the supernatants. The protein concentration was quantified using the Bio-Rad protein assay. The protein samples (20–40 μg/lane) were resolved by electrophoresis on 10% sodium dodecyl sulfate-polyacrylamide gels prior to being transferred to a PVDF membrane as previously described [20]. Blots were blocked by incubation with 5% skimmed dry milk in TBST (50 mM Tris·HCl, 100 mM NaCl and 0.1% Tween 20 at pH 7.5) for 1 h at room temperature. Subsequently, blots were incubated overnight at 4 °C with rabbit anti-phosphorylated IκBα (Ser32) and rabbit anti-IκBα (dilution, 1:800–1,000; Cell Signaling Technology, Inc., Danvers, MA, USA) in blocking solution. Following incubation with the primary antibodies, blots were washed and incubated with bovine anti-rabbit horseradish peroxidase-conjugated secondary antibody (dilution, 1:1,000-2,000; Santa Cruz Biotechnology, Santa Cruz, CA, USA) for 1 h at room temperature. Detection was accomplished using an enhanced chemiluminescence Western blot test kit (Amersham Biosciences, Piscataway, NJ, USA). Band intensity was densitometrically assayed. β-Actin was used to normalize protein loaded on blots.

### NF-κB p65 DNA binding activity assay

A total of 30 min after treatment, nuclear protein was isolated from the cells using a nuclear extract kit (Active Motif, Carlsbad, CA, USA), according to the manufacturer’s protocol. Protein concentrations of nuclear extract were determined using a protein assay kit. The DNA binding activity of NF-κB p65 was evaluated using a non-radioactive, ELISA-based TransAM NF-κB p65 assay kit (Active Motif, Carlsbad, CA, USA), according to the manufacturer’s protocol. The DNA binding activity of NF-κB p65 was normalized to the concentration of nuclear protein.

### Dual-luciferase reporter assay for NF-κB

To determine NF-κB transcriptional activity, HUVECs were transfected with a cis-reporter plasmid containing 5 copies of consensus NF-κB sequences linked to a minimal E1B promoter-luciferase gene (pNF-κB Luc; Stratagene, LaJolla, CA, USA) using LipofectAMINE 2000, according to the manufacturer’s protocol. pRL-SV40 *Renilla* luciferase control reporter vector (Promega Corporation, Madison, WI, USA) was co-transfected as an internal control. A total of 24 h after transfection, the cells were treated with LPS in the presence or absence of triptolide at the indicated doses. After 1 h, the cells were harvested and lysed with cell lysis buffer (Cell Signaling Technology, Inc., Danvers, MA, USA). Firefly luciferase activity was measured using a Dual-Luciferase Reporter Assay System (Promega Corporation, Madison, WI, USA) with a TD-20/20 luminometer (Turner BioSystems, Sunnyvale, CA, USA). Relative luciferase activity is presented as a ratio of firefly luciferase activity to *Renilla* luciferase activity.

### Statistical analysis

All experiments were performed independently at least four times and the results are presented as the mean ± standard error of the mean. The differences between untreated groups and treated groups were determined using one-way analysis of variance, followed by a Bonferroni’s adjustment for multiple comparisons. *p* < 0.05 was considered to indicate a statistically significant difference. All analyses were performed using GraphPad Prism 6.0 software (GraphPad Software, Inc., La Jolla, CA, USA).

## Results

### Triptolide decreases the viability of HUVECs

**Fig. 1:**
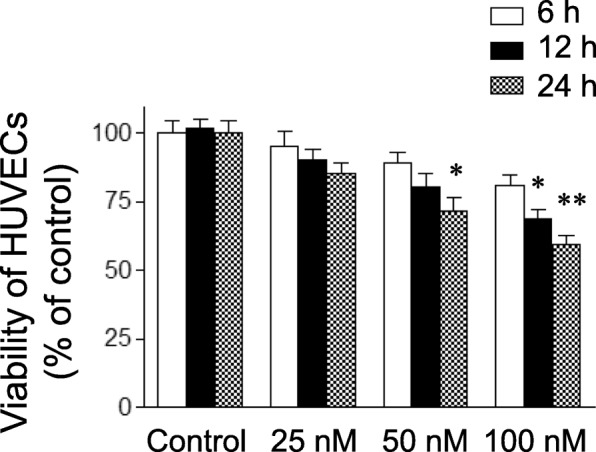
The effects of triptolide on the viability of human umbilical vein endothelial cells (HUVECs). HUVECs were treated with triptolide (25, 50 or 100 nM) for the indicated times. Cell viability was determined by an MTT assay. Data are expressed as the mean ± standard error of the mean. *p < 0.05 and **p < 0.01, compared with the corresponding control group

Triptolide decreased the viability of HUVECs in a dose- and time-dependent manner (Fig. 1). Compared with the control group, the viability of HUVECs was decreased after 12 or 24 h of exposure to triptolide at a concentration of 100 or 50 nM, respectively. However, the cell viability did not change when HUVECs were exposed to triptolide at concentrations of 25, 50 or 100 nM for 6 h. In order to analyze the events more fully, triptolide was used at the doses of 25, 50 and 100 nM for 6 h in the present study. In addition, previous studies by our laboratory have demonstrated that treatment with LPS (1 μg/ml) for 6 h had no effect on the viability of HUVECs [20].

### Triptolide attenuates LPS-induced production of pro-inflammatory cytokines and chemokines in HUVECs

**Fig. 2:**
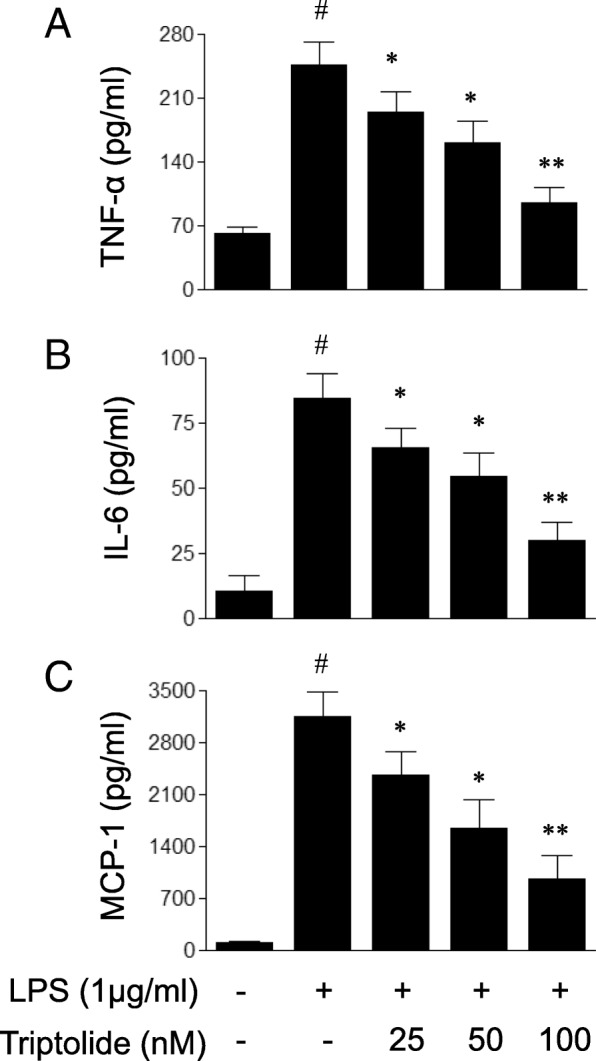
The effects of triptolide on lipopolysaccharide (LPS)-induced production of pro-inflammatory (**a**) TNF-α, (**b**) IL-6 and (**c**) MCP-1 in human umbilical vein endothelial cells (HUVECs). HUVECs were treated with LPS (1 μg/ml) in the presence or absence of triptolide (25, 50 or 100 nM) for 6 h. The ELISA assay was used to measure the concentrations of pro-inflammatory cytokines and chemokines in the culture medium. Data are expressed as the mean ± standard error of the mean. *p < 0.05 and **p < 0.01, compared with LPS treatment alone. #*p* < 0.05, compared with the control group

To assess the possible impact of triptolide on endothelial cell-associated inflammatory responses, the present study first determined the effects of triptolide on the LPS-induced production of inflammatory mediators in endothelial cells. As demonstrated in Fig. 2, treatment with LPS (1 μg/ml) for 6 h increased the production of pro-inflammatory cytokines and chemokines, including TNF-α, IL-6 and MCP-1 in HUVECs. The LPS-induced effects on pro-inflammatory cytokines and chemokines were dose-dependently inhibited by pre-treatment with triptolide 1 h prior to LPS treatment. Taken together, the results of the present study demonstrated that triptolide suppresses inflammatory responses in endothelial cells.

### Triptolide attenuates LPS-induced expression of adhesion molecules in HUVECs

**Fig. 3:**
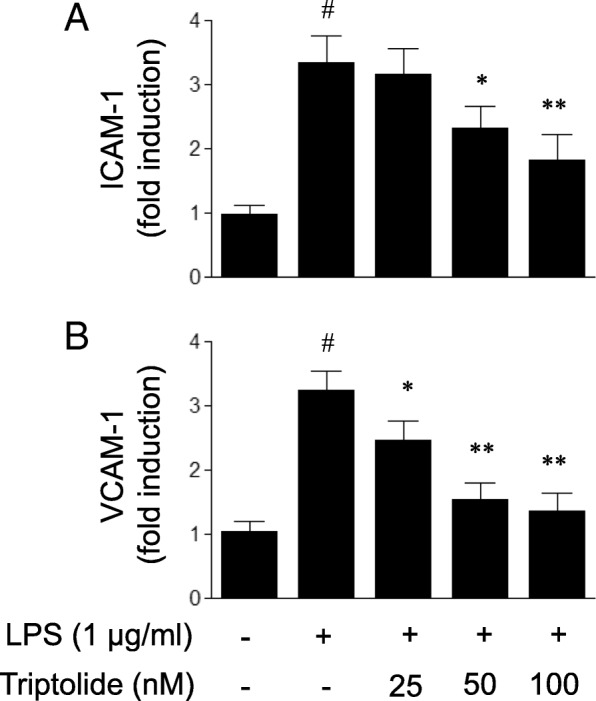
The effects of triptolide on lipopolysaccharide (LPS)-induced (**a**) ICAM-1 and (**b**) VCAM-1 expression in human umbilical vein endothelial cells (HUVECs). HUVECs were treated with LPS (1 μg/ml) in the presence or absence of triptolide (25, 50 or 100 nM) for 6 h. The cell-based ELISA assay was used to quantify the expression of ICAM-1 and VCAM-1 in HUVECs. Data are expressed as the mean ± standard error of the mean. *p < 0.05 and **p < 0.01, compared with LPS treatment alone. #*p* < 0.05, compared with the control group

It has been recognized that adhesion molecules are responsible for the pathological process of leukocyte infiltration. Therefore, it was subsequently examined whether triptolide treatment inhibits the LPS-induced expression of adhesion molecules in endothelial cells. As illustrated in Fig. 3, LPS (1 μg/ml) treatment evoked an increase in ICMA-1 and VCAM-1 expression in HUVECs, and pre-treatment with triptolide was revealed to attenuate the LPS-induced expression of ICAM-1 and VCAM-1 in a dose-dependent manner. Consequently, the diminished adhesion molecule expression induced by triptolide may contribute to attenuating the adhesion of leukocytes to endothelial cells.

### Triptolide attenuates LPS-induced monocyte adhesion to HUVECs

**Fig. 4:**
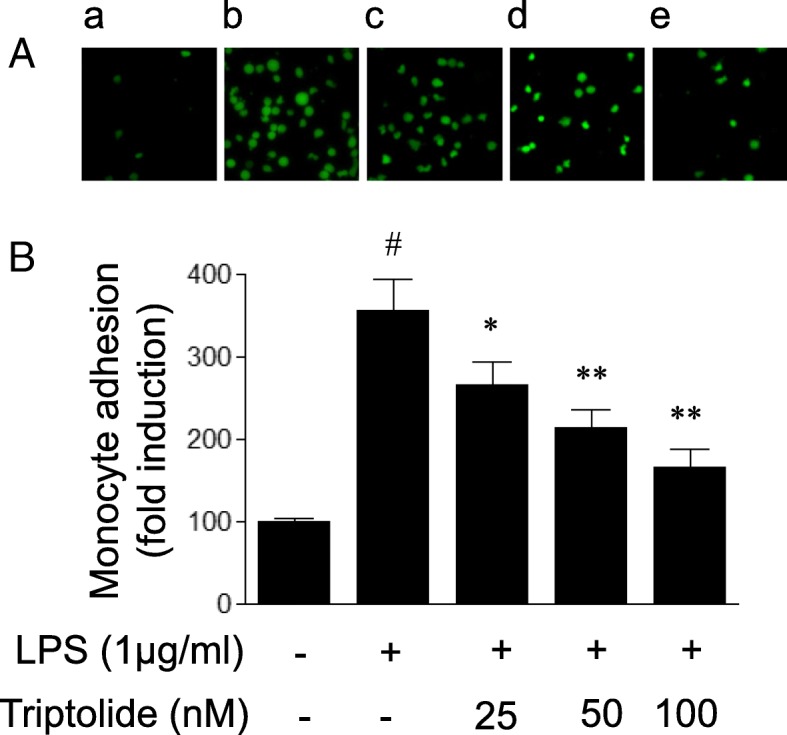
The effects of triptolide on lipopolysaccharide (LPS)-induced monocyte adhesion to human umbilical vein endothelial cells (HUVECs). HUVECs were treated with LPS (1 μg/ml) in the presence or absence of triptolide (25, 50 or 100 nM) for 6 h, and subsequently co-cultured with fluorescence-labeled THP-1 cells for 30 min. (**a**) Representative images of fluorescence staining of monocyte adhesion to HUVECs. Magnification, × 200. HUVECs were exposed to (a) vehicle (control), (b) LPS or LPS with triptolide at the concentration of (c) 25, (*d*) 50 or (*e*) 100 nM prior to incubation with fluorescence-labeled THP-1 cells. (**b**) The adhesion ability of THP-1 cells was assayed using a spectrofluorometer. Data are expressed as the mean ± standard error of the mean. *p < 0.05 and **p < 0.01, compared with LPS treatment alone. #*p* < 0.05, compared with the control group

Since attachment of leukocytes to endothelial cells via adhesion molecules is a central step in the development of inflammation, the present study determined whether triptolide inhibits leukocyte adhesion to endothelial cells. As demonstrated in Fig. 4, LPS (1 μg/ml) treatment for 6 h increased the number of monocytes that attached to HUVECs. Pretreatment with triptolide dose-dependently inhibited LPS-induced monocyte adhesion. Taken together, these results suggested that triptolide inhibits LPS-induced leukocyte adhesion to endothelial cell possibly via suppression of adhesion molecule expression.

### Triptolide attenuates LPS-induced activation of NF-κB pathway in HUVECs

**Fig. 5:**
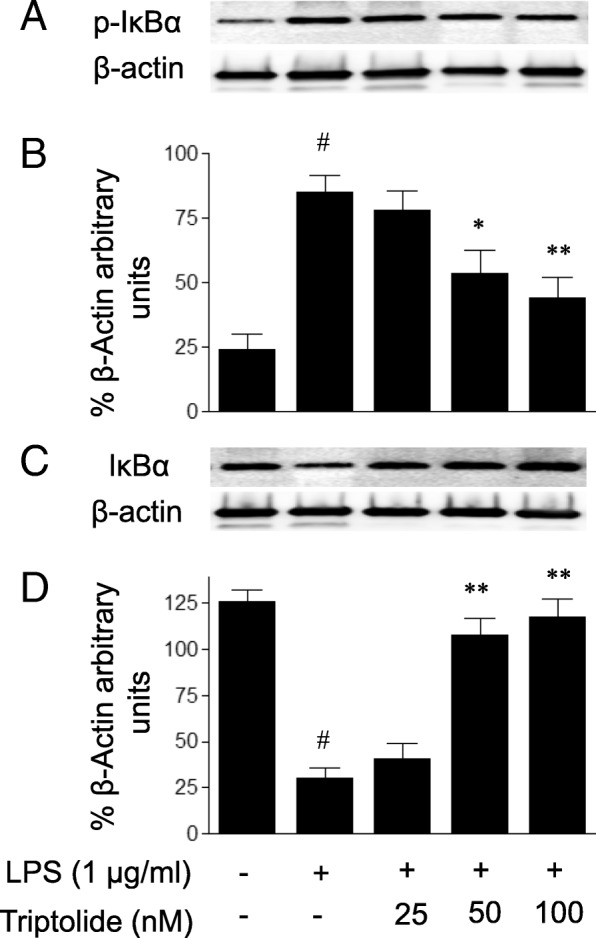
The effects of triptolide on lipopolysaccharide (LPS)-induced changes in the protein levels of (**a** and **b**) phosphorylated IκBα (p-IκBα) and (**c** and **d**) IκBα in human umbilical vein endothelial cells (HUVECs). Full-length blots for p-IκBα and IκBα are presented in Additional file Data. HUVECs were treated with LPS (1 μg/ml) in the presence or absence of triptolide (25, 50 or 100 nM) for 30 min. Cell lysates were subjected to immunoblot analysis using the antibodies against p-IκBα (Ser32) and IκBα. The data are presented as the relative protein expression normalized to β-actin expression. Data are expressed as the mean ± standard error of the mean. *p < 0.05 and **p < 0.01, compared with LPS treatment alone. #*p* < 0.05, compared with the control group

**Fig. 6:**
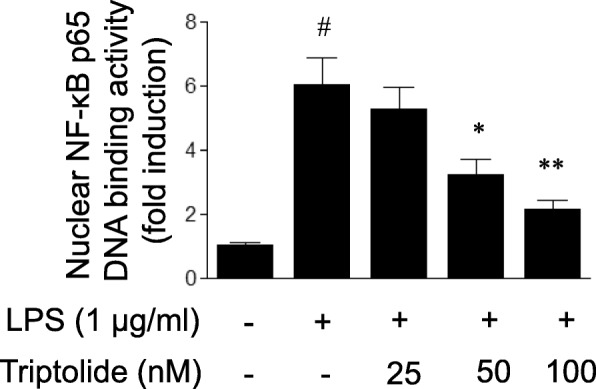
The effects of triptolide on lipopolysaccharide (LPS)-induced increases in NF-κB p65 DNA binding activity in human umbilical vein endothelial cells (HUVECs). HUVECs were treated with LPS (1 μg/ml) in the presence or absence of triptolide (25, 50 or 100 nM) for 30 min. The DNA binding activity of NF-κB p65 in HUVECs was determined using a non-radioactive, ELISA-based assay kit. Data are expressed as the mean ± standard error of the mean. *p < 0.05 and **p < 0.01, compared with LPS treatment alone. #*p* < 0.05, compared with the control group

**Fig. 7:**
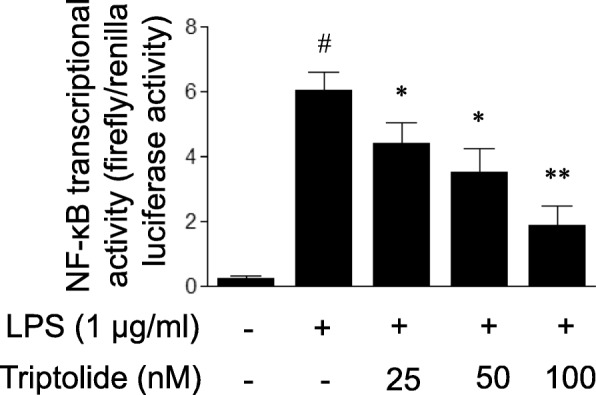
The effects of triptolide on lipopolysaccharide (LPS)-induced NF-κB transcriptional activation in human umbilical vein endothelial cells (HUVECs). HUVECs were treated with LPS (1 μg/mL) in the presence or absence of triptolide (25, 50, or 100 nM) for 1 h. The NF-κB transcriptional activity in HUVECs was determined using a dual luciferase reporter assay system as described in the method section. The data were presented as a ratio of firefly to Renilla luciferase activities. Data are expressed as the mean ± standard error of the mean. *p < 0.05, **p < 0.01 compared with LPS treatment alone. #*p* < 0.05 compared with control group

NF-κB is a crucial transcriptional factor for the expression of inflammatory mediators. The phosphorylation of IκBα (Ser32) and its degradation are required for the binding of NF-κB to DNA. Therefore, the present study examined the effect of triptolide on LPS-induced NF-κB activation by measuring IκBα (Ser32) phosphorylation. As demonstrated in Fig. 5, LPS treatment for 30 min led to the phosphorylation of IκBα in HUVECs, and the responses were attenuated by pre-treatment with triptolide. Additionally, the LPS-induced degradation of IκBα was attenuated by triptolide pre-treatment. Full-length Western blots for the protein expression of IκBα phosphorylation and IκBα are presented in Additional file 1: Figures S1 and S2. Since the phosphorylation of IκBα and its degradation are followed by nuclear NF-κB DNA binding and transcriptional activation, the effect of triptolide on LPS-induced NF-κB activation was subsequently determined by assaying the DNA binding activity of NF-κB p65 and NF-κB transcriptional activity, respectively. As demonstrated in Fig. 6, the DNA binding activity of NF-κB p65 was markedly increased following LPS treatment in HUVECs, and the LPS-induced binding of NF-κB to DNA was attenuated by pre-treatment with triptolide in a dose-dependent manner. Furthermore, treatment with LPS led to increased NF-κB reporter activity in HUVECs and this response was dose-dependently inhibited by pre-treatment with triptolide (Fig. 7).

Taken together, the results of the present study showed that triptolide treatment suppressed LPS-induced NF-κB transcriptional activation with the inhibition of IκBα phosphorylation and its degradation, and subsequent NF-κB DNA binding.

## Discussion

The present study provides the first characterization of the anti-inflammatory actions of triptolide in endothelial cells. To begin with, it was demonstrated that triptolide, a major bioactive component of the Chinese herb TWHF, leads to an anticipated inhibition of endothelial inflammatory response induced by LPS. The anti-inflammatory effects include decreased release of pro-inflammatory cytokines and chemokines, and decreased expression of adhesion molecules that contribute to modulating inflammatory cell adhesion to endothelial cells. Next, mechanistic analysis revealed that the anti-inflammatory effect of triptolide is mediated possibly via suppression of NF-κB activation. Therefore, it is the first study to show that triptolide attenuates endothelial cell-associated inflammatory responses possibly via inhibition of NF-κB signaling pathway.

Triptolide is a major bioactive compound in Traditional Chinese Medicine TWHF, which has been widely used as a therapeutic agent for hundreds of years in China [6–8, 21]. Despite considerable advances in in vivo research for triptolide in the field of anti-inflammation, it is unclear whether it has anti-inflammatory effects on vascular endothelial cells. Given that endothelial cells serve a pivotal role in inflammation by the release and expression of inflammatory mediators and adhesion molecules that recruit leukocytes from blood to tissues [22], the present study assessed the ability of triptolide to modulate the release of inflammatory mediators and activation of the NF-κB signaling pathway in LPS-stimulated HUVECs. The results of the present study demonstrated that triptolide attenuated LPS-induced endothelial inflammatory responses. Notably, these effects were associated with suppression of NF-κB activation. Taken together, the results suggested that triptolide attenuates inflammatory responses possibly via inhibition of NF-κB activation.

The integrated function of endothelial cells serves a crucial role in the maintenance of cardiovascular homeostasis [23]. Pro-inflammatory stimuli, including LPS and TNF-α, usually cause endothelial dysfunction, leading to the initiation of inflammatory responses in numerous cardiovascular diseases [3, 4, 20]. Inflammatory responses occurring in endothelial cells is featured by the overproduction of inflammatory mediators, including pro-inflammatory cytokines (TNF-α and IL-6), chemokines (MCP-1) and adhesion molecules (ICAM-1 and VCAM-1). Activation of the NF-κB signaling pathway contributes to the expression of inflammatory genes in endothelial cells [24]. Therefore, it is well accepted that modulation of the processes is an effective strategy for the treatment of the diseases characterized with inflammation.

TNF-α, IL-6 and MCP-1 are important pro-inflammatory cytokines or chemokines. They are well characterized inflammatory mediators, and are involved in the initiation and amplification of inflammatory responses. The present study selected TNF-α, IL-6 and MCP-1 as biomarkers of endothelial inflammatory responses, since there is a strong correlation between the release and expression of these inflammatory mediators and cardiovascular morbidity and mortality [25, 26]. In addition, there is accumulating evidence demonstrating that these inflammatory mediators serve a key role in the development of cardiovascular diseases [27–29]. Although we only determined the effects of triptolide on these inflammatory mediators, it is reasonable to propose that triptolide may also work on other inflammatory mediators. Therefore, further studies are required to clarify this issue.

Inflammation is the body’s protective mechanism against infection or injury. However, when the inflammatory response becomes severe or prolonged, it leads to a pathological state such as sepsis and atherosclerosis. In addition to the production of inflammatory mediators, inflammation is characterized by massive recruitment of leukocytes from the blood into the inflammatory sites. The infiltration of leukocytes involves the interaction between adhesion molecules on endothelial cell surfaces and its counter receptors on the surface of leukocytes [22]. Chemokines (e.g., MCP-1) are also involved in recruiting leukocytes to sites of inflammation. The expression of the inflammatory mediators is under the tight control of the inflammatory transcription factor NF-κB [24].

A characterization of the effects of triptolide on NF-κB activation in endothelial cells was determined. It is well known that NF-κB is activated upon phosphorylation of its cytoplasmic inhibitor IκBα by IκB kinase (IKK) complex [30, 31]. IKK-induced phosphorylation triggers the ubiquitination-mediated degradation of IκBα and subsequent nuclear NF-κB DNA binding, leading to the transcription of inflammatory genes. To delineate the mechanisms by which triptolide regulates NF-κB activity, unrelated methodological approaches were used. As expected, the present study demonstrated that triptolide, in addition to inhibiting NF-κB transcriptional activation as measured with an NF-κB luciferase reporter gene assay, also suppressed the events upstream of NF-κB transcriptional activation, including IκBα phosphorylation and its degradation, and NF-κB DNA binding. The results implied that triptolide attenuates LPS-induced NF-κB transcriptional activation at a cytosolic and nuclear site.

Limitations of this study include the focus of our experiments in specific endothelial cell lines. The characterization of these responses to primary endothelial cells from different tissues remains to be determined. Furthermore, in addition to LPS, other pro-inflammatory stimuli, including oxidized low-density lipoprotein (ox-LDL) and TNF-α, also cause endothelial dysfunction, resulting in the initiation of inflammatory responses in numerous cardiovascular diseases [3, 4, 32]. Therefore, the effects of triptolide on endothelial cells exposed to other pro-inflammatory stimuli (e.g., ox-LDL) will be determined in the future studies.

## Conclusions

In summary, the results of the present study demonstrated that triptolide possesses anti-inflammatory effects on inflammatory responses in endothelial cells. Moreover, to the best of our knowledge, the present study was the first to show that the mechanisms for suppression of endothelial cell-associated inflammatory responses by triptolide may involve inhibition of the NF-κB signaling pathway.

Inflammation is the most common characteristic of a number of diseases, including atherosclerosis and hypertension, and there is a clear requirement for effective therapies. The results of the present study provided insight into the mechanisms for inhibition of endothelial cell-associated inflammatory responses by triptolide, a major bioactive component of the Chinese herb TWHF, and will contribute to our understanding of the mechanisms by which TWHF and its preparations attenuate inflammatory responses.

## Additional file

Additional file 1: Supplementary Data. **Figure S1.** Full-length Western blots for Figure 5A, the effects of triptolide on lipopolysaccharide (LPS)-induced changes in the protein levels of phosphorylated IκBα (p-IκBα) in human umbilical vein endothelial cells (HUVECs). The blots show p-IκBα (A) with blots stripped and reprobed to detect β-actin (B). The blots are presented individually for each staining, intact without splicing. **Figure S2.** Full-length Western blots for Figure 5C, the effects of triptolide on lipopolysaccharide (LPS)-induced changes in the protein levels of IκBα in human umbilical vein endothelial cells (HUVECs). The blots show IκBα (A) with blots stripped and reprobed to detect β-actin (B). The blots are presented individually for each staining, intact without splicing. (DOCX 705 kb)

## Data Availability

The datasets used and/or analyzed during the current study are available from the corresponding author on reasonable request.

## Abbreviations

DMSO: dimethyl sulfoxide
EBM-21: endothelial basal medium-2
FBS: fetal bovine serum
HUVECs: human umbilical vein endothelial cells;
IKK: IκB kinase
LPS: lipopolysaccharide
MTT: 3-(4,5-dimethylthiazol-2-yl)-2,5-diphenyltetrazolium bromide
NF-κB: nuclear factor-κB
ox-LDL: oxidized low-density lipoprotein
PVDF: polyvinylidene difluoride
TWHF: *Tripterygium wilfordii* Hook.F.
