# Supplementary material for: Triptolide attenuates lipopolysaccharide-induced inflammatory responses in human endothelial cells: involvement of NF-κB pathway
Source: BMC Complement Altern Med. 2019 Aug 2;19:198. doi: 10.1186/s12906-019-2616-3 (PMC6679459; doi:10.1186/s12906-019-2616-3)
Supplement: Supplementary file 1 — Supplementary Data. Figure S1. Full-length Western blots for Figure 5A, the effects of triptolide on lipopolysaccharide (LPS)-induced changes in the protein levels of phosphorylated IκBα (p-IκBα) in human umbilical vein endothelial cells (HUVECs). The blots show p-IκBα (A) with blots stripped and reprobed to detect β-actin (B). The blots are presented individually for each staining, intact without splicing. Figure S2. Full-length Western blots for Figure 5C, the effects of triptolide on lipopolysaccharide (LPS)-induced changes in the protein levels of IκBα in human umbilical vein endothelial cells (HUVECs). The blots show IκBα (A) with blots stripped and reprobed to detect β-actin (B). The blots are presented individually for each staining, intact without splicing. (DOCX 705 kb) [file 12906_2019_2616_MOESM1_ESM.docx]

MS#: BCAM-D-19-00700R3

**Triptolide attenuates lipopolysaccharide-induced inflammatory responses in human endothelial cells: Involvement of NF-κB pathway**

Chundong Song^*^, Youping Wang^*, §^, Lin Cui, Fengna Yan, Si Shen

*Central Laboratory and Department of Pediatrics, Division of Cardiology, First Affiliated Hospital, Henan University of Traditional Chinese Medicine, Zhengzhou, 450000, China.*

^*^*C. Song and Y. Wang contributed equally to this work.*

**^§^ Corresponding author:**

*Youping Wang, M.D., Ph.D.*

*Central Laboratory and Division of Cardiology,*

*First Affiliated Hospital, Henan University of Traditional Chinese Medicine,*

*Zhengzhou, 450000, China.*

*Phone: +86-371-66248345*

*E-mail: [wangyp8@163.com](mailto:wangyp8@163.com)*

**E-mail addresses for all authors:**

*C. Song:* [*scd670918@126.com*](mailto:scd670918@126.com)

*Y. Wang:* [*wangyp8@163.com*](mailto:wangyp8@163.com)

*L. Cui:* [*henantcm@126.com*](mailto:henantcm@126.com)

*F. Yan:* [*m18239211525@163.com*](mailto:m18239211525@163.com)

*S. Shen:* [*d547395571@126.com*](mailto:d547395571@126.com)

**Supplementary Data**

**Results**

Supplementary Figure S1

Figure S1. Full-length Western blots for Figure 5A, the effects of triptolide on lipopolysaccharide (LPS)-induced changes in the protein levels of phosphorylated IκBα (p-IκBα) in human umbilical vein endothelial cells (HUVECs). The blots show p-IκBα (A) with blots stripped and reprobed to detect β-actin (B). The blots are presented individually for each staining, intact without splicing.

Supplementary Figure S2

Figure S2. Full-length Western blots for Figure 5C, the effects of triptolide on lipopolysaccharide (LPS)-induced changes in the protein levels of IκBα in human umbilical vein endothelial cells (HUVECs). The blots show IκBα (A) with blots stripped and reprobed to detect β-actin (B). The blots are presented individually for each staining, intact without splicing.
